# Tracking macrophages by direct and indirect ^89^Zr PET imaging

**DOI:** 10.1007/s00259-025-07752-8

**Published:** 2026-02-13

**Authors:** Vittorio De Santis, Renee Flaherty, Arshiya Banu, Paul Gape, Sophie Langdon, Alex Rigby, Aishwarya Mishra, George Firth, Truc T. Pham, Amaia Carrascal-Miniño, Jana Kim, Kavitha Sunassee, Matthew Cleveland, Rafael T. M. De Rosales, Timothy H. Witney, Michelle T. Ma, Samantha Y. A. Terry

**Affiliations:** 1 Department of Imaging Chemistry and Biology, School of Biomedical Engineering and Imaging Sciences, King’s College London, London, SE1 7EH UK; 2 Division of Breast Cancer Research, The Breast Cancer Now Toby Robins Research Centre, The Institute of Cancer Research, London, SW3 6JB UK; 3 GSK Medicines Research Centre, Gunnels Wood Road, Stevenage, Hertfordshire SG1 2NY UK

**Keywords:** Macrophages, Cell tracking, Positron emission tomography, F4/80, Oxine

## Abstract

**Purpose:**

Tumour-associated macrophages are highly abundant in the tumour microenvironment, and their levels influence tumour progression and therapy response. Tracking macrophages by positron-emission tomography (PET) can yield critical information on the dynamics of macrophage infiltration, particularly in diseases such as breast cancer, in which tumour-associated macrophages are significant in the evolution of the tumour microenvironment.

**Methods:**

Two complementary PET imaging methods were used to track macrophages. The first method used a novel [^89^Zr]Zr-labelled antibody that targets the murine F4/80 receptor, which is located on the cell surface of *in situ* macrophages. [^89^Zr]Zr-DFO-anti-F4/80 and a non-specific [^89^Zr]Zr-labelled control antibody were developed and characterised for their specificity. The second method involved directly labelling mouse-derived macrophages with [^89^Zr]Zr-oxine, and the effect of [^89^Zr]Zr-radiolabelling on macrophage viability and phenotype was evaluated. Using PET imaging and *ex vivo* tissue counting, the in vivo biodistribution of [^89^Zr]Zr-DFO-anti-F4/80 and [^89^Zr]Zr-labelled murine macrophages was quantified in female BALB/c mice bearing syngeneic, orthotopic 4T1 breast cancer tumours.

**Results:**

[^89^Zr]Zr-DFO-anti-F4/80 showed target-specific uptake *in vitro*. At a dose of 100 μg DFO-anti-F4/80 per mouse, [^89^Zr]Zr-DFO-anti-F4/80 enabled visualization of tumour-resident macrophages by PET/CT with high contrast. This was not observed for non-specific [^89^Zr]Zr-DFO-IgG2b. PET/CT imaging showed that intravenously administered [^89^Zr]Zr-labelled M0 macrophages migrated to the liver and spleen, with a proportion of macrophages trafficking to the tumour. [^89^Zr]Zr-oxine labelling did not affect macrophage viability nor phenotype, even at an absorbed radiation dose of 3.31 Gy.

**Conclusion:**

For the first time, we have applied and compared two complementary PET/CT imaging strategies to quantify the infiltration of macrophages in an orthotopic, syngeneic murine model of breast cancer. First, PET/CT imaging using an “indirect” cell tracking approach with a new [^89^Zr]Zr-DFO-anti-F4/80 antibody radiotracer showed that resident tumour-associated macrophages are abundant in this syngeneic breast cancer model. Second, PET/CT imaging using [^89^Zr]Zr-labelled M0 macrophages showed that macrophages in the blood pool migrate to the tumour, within 24 h. These tools have utility in quantifying macrophage distribution and migration in cancer, particularly in the context of new and existing therapies in which macrophage populations are perturbed.

**Supplementary Information:**

The online version contains supplementary material available at 10.1007/s00259-025-07752-8.

## Introduction

Macrophages are crucial mediators of the innate immune system. However, their function can become dysregulated in a range of diseases, including respiratory disorders, and inflammatory diseases such as rheumatoid arthritis and cancer [1–5]. Tumour-associated macrophages (TAMs) play a critical role in tumour growth, cell migration, invasion, and metastasis in multiple tumour types, including in breast cancer [6–9]. TAMs can also prime the pre-metastatic niche and mediate therapeutic resistance [7, 10, 11]. As such, high TAM levels can correlate with poor prognoses in solid tumours and a range of therapeutic strategies are in development to modulate their activity [12]. Such approaches include decreasing TAM populations within tumours by blocking monocyte recruitment, priming anti-tumour phagocytic/inflammatory phenotypes, gene silencing with small interfering RNA-based drugs, or the use of immunotherapies [13].

The ability to image and quantify the biodistribution of TAMs, including the recruitment of TAMs within tumours, has the potential to revolutionise TAM-targeted therapies and elucidate the determinants of aberrant macrophage biology during tumour development. Existing methods to evaluate TAMs include immunohistochemistry, gene analysis, flow cytometry, and imaging with ultrasound (CSF-1R- or folate-conjugated nanobubbles [14–17]), fluorescence (cyanine 7-labelled deoxymannose or dye-anti-CD206 [14–17]), or magnetic resonance (MR; iron oxide-nanoparticles [18, 19]). Radionuclide single photon emission computed tomography (SPECT) and positron emission tomography (PET) modalities have also been applied to macrophage imaging (*vide infra*).

Broadly, PET or SPECT imaging of immune cells involves either (i) directly radiolabelling and tracking administered immune cells, or (ii) using a radiotracer that targets receptors expressed on the surface of the *in situ* immune cell. “Direct” radiolabelling methods mostly use radiometal-ionophore complexes, e.g. [^89^Zr]Zr-oxine (PET) and [^111^In]In-oxine (SPECT/γ-scintigraphy), to radiolabel and then track white blood cells, T cells, and natural killer cells post intravenous injection [20–23]. In the second “indirect” approach, an administered radiotracer targets a receptor selectively expressed on the cell surface of the immune cell. There is a range of receptor-targeted radiotracers that have been developed for imaging *in situ* macrophages, namely via macrophage surface markers or radiolabelled particles [24–31]. Importantly, several TAM receptor-targeted radiopharmaceuticals have been evaluated in clinical oncology studies, underscoring the need of quantitative TAM imaging. For example, tracers targeting TAMs via the translocator protein TSPO showed that the degree of tracer localisation correlated with tumour grade and mutation status [32–35]. Other TAM tracers are in clinical development, for example, through the imaging of the mannose receptor in breast cancer and melanoma using [^68^ Ga]Ga-NOTA-anti-MMR-VHH2 PET (NCT04168528).

Despite the available options, there remains a need for quantitative imaging, not only of local macrophage distribution but also to assess macrophage migration. By combining both direct and indirect immune cell imaging methods, we aim to obtain these complementary insights.

Here, for the first time, we have developed and applied *both* direct and indirect macrophage PET imaging methods in a murine syngeneic, orthotopic breast cancer model to obtain an overview of macrophage residency and migration into the tumour microenvironment. Given that a spectrum of activated macrophage states is reported in breast cancer patients [36], we utilised pan-macrophage approaches to investigate TAMs within our model. Building on our previous work using pan-macrophage tracer [^111^In]In-DTPA-anti-F4/80 [37], [^89^Zr]Zr-DFO-anti-F4/80 was developed to image endogenous murine macrophage populations. Separately, murine macrophages were also directly labelled *ex vivo* with [^89^Zr]Zr-oxine to enable longitudinal tracking of the migration of exogenous macrophage migration and distribution. This powerful strategy combines two complementary methods to enable quantitative radionuclide imaging of macrophage distribution and dynamics in a cancer model.

## Materials and methods

### Antibody conjugation, radiolabelling and quality control

Anti-F4/80 and irrelevant antibody control IgG2b were incubated with p-isothiocyanatobenzyl-desferrioxamine (p-NCS-Bz-DFO in DMSO) at a 10:1 molar equivalent ratio of p-NCS-Bz-DFO:antibody at 37 °C for 30 min followed by 16-h incubation at room temperature (RT). DFO-anti-F4/80 or DFO-IgG2b were then purified. For further details, see Supplementary Methods.

For antibody radiolabelling, [^89^Zr]Zr-oxalate (26.7 MBq in 250 µL, Perkin Elmer) was ion-exchanged to [^89^Zr]ZrCl_4_ using Sep-Pak® QMA-light cartridges (Waters) as described previously [38], with recovery yields of 88.4% ± 5.8%. Liquid was evaporated at 100 °C under a nitrogen stream before reconstituting the contents to 1 MBq/10 µL in 0.2 M HEPES (pH 7). DFO-anti-F4/80 and DFO-IgG2b (1 mg) were then incubated with [^89^Zr]ZrCl_4_ (26 MBq) at a 1:1 volumetric ratio of antibody to [^89^Zr]ZrCl_4_ in HEPES solution at 37 °C for 1 h. Radiolabelled antibodies were then purified into 0.1 M ammonium acetate (pH 5.5) by a PD10 column. Radio-HPLC analysis using a Phenomenex BioSep SEC-S2000 column (Agilent Technologies) in 2 mM EDTA in PBS was carried out for radiolabelled antibodies before and after PD10 purification.

### Bone marrow-derived macrophages and their staining

Female BALB/c mice (Charles River/Envigo) at 6–9 weeks were humanely killed by rising concentration of CO_2_ for dissection of intact femurs and tibias from which the bone marrow-derived monocytes (BMDMs) were isolated and differentiated *ex vivo* to M0 macrophages (see Supplementary Methods). Secondary death confirmation was by cervical dislocation. M0 macrophages, grown on coverslips, were stained for ionised calcium binding adaptor molecule 1 (IBA-1) and F4/80 for immunofluorescence imaging and analysed by flow cytometry for F4/80 expression. For further details, see Supplementary Methods and Figure S1.

### [^89^Zr]Zr-oxine synthesis and quality control

An oxine kit was produced as previously described [21]. To 100 µL oxine kit (50 µg oxine, pH 7.9–8), 18 µL [^89^Zr]Zr-oxalate (Perkin Elmer) was added for 15 min at RT. Instant thin layer chromatography (iTLC) was carried out for quality control using Whatman paper n1 with a mobile phase of ethyl acetate. Radiolabelling efficiency was analysed using a Scan-RAM (Lablogic Systems)/Mini-Scan™ radioTLC linear scanner (Eckert & Ziegler medical) and Laura software (Lablogic Systems).

### Cancer cell culture

4T1 murine breast cancer cells (ATCC) were cultured in RPMI-1640 supplemented with 10% fetal bovine serum and 100 U/mL penicillin and 100 µg/mL streptomycin (Invitrogen) and 2 mM L-glutamine (Invitrogen) and maintained at 37 °C in a humidified atmosphere with 5% CO_2_.

### Uptake and efflux assays

For antibody uptake assays, fresh BMDM (1 × 10^6^) or 4T1 cells (2 × 10^5^) were plated in 6-well plates 24 h prior to uptake assays. 5 kBq of [^89^Zr]Zr-DFO-anti-F4/80 (~ 1 ng) was added to cells in the presence or absence of a blocking dose of unlabelled antibody (10 μg) for 1 h at 37 °C. Cells were then washed, lysed in RIPA lysis buffer, and the radioactivity in the lysates measured in a gamma counter (LKB Wallac, CompuGamma). Data was expressed as the percentage of radioactivity bound compared to the total amount added to cells.

For [^89^Zr]Zr-oxine uptake assays, M0 macrophages, either in suspension (100 µL) or adhered (500 µL), were incubated with [^89^Zr]Zr-oxine (300 kBq, 1.9 µL and 0.9 µg of oxine) in serum-free RPMI-1640 for 1 h before washing, trypsinising (if grown adherent) and centrifuging at 500 × *g*. Radioactivity in the pellets and supernatants was counted using a Wallac Wizard gamma counter. For efflux assays, cells were washed following 1 h of incubation and the amount of radioactivity released into the medium was measured over time.

### Cellular dosimetry

Uptake and efflux data was used for cellular dosimetry (see Supplementary Methods) using a 2-compartment model to represent distribution of activity in labelling and incubation phases (Figure S2).

### [^89^Zr]Zr-oxine effect on macrophage polarisation and viability

Adherent M0 macrophages (2 × 10^6^ cells/well) in RPMI-1640 medium supplemented with 10 ng/mL mM-CSF were incubated with 600 kBq [^89^Zr]Zr-oxine at 37 °C for 15 min before removing excess [^89^Zr]Zr-oxine and washing with PBS. Macrophages were then fixed in 4% paraformaldehyde for 10 min, before being stained and analysed for F4/80 (pan macrophage marker), CD80 (M1 macrophage marker), and CD206 (M2 macrophage marker) as described in Supplementary Methods.

Viability of adherent or suspension M0 macrophages was determined using the Trypan blue dye exclusion assay at 1, 2, 24, 48 and 72 h post radiolabelling with [^89^Zr]Zr-oxine.

### Tumour inoculation and maintenance

Female BALB/c mice (6–9 weeks, Charles River) were anaesthetised using 2–2.5% isoflurane in 100% oxygen and 5 × 10^5^ 4T1 cells in 100 µL PBS were injected into the mammary fat pad. Mice were then recovered, and mouse weight and tumour growth monitored. Once tumours reached approximately 100 mm^3^, as measured by calliper and using Volume = 0.5 × Length × Width^2^ (~ 12 days post inoculation), mice were matched for tumour size and imaged, or tumours were harvested for flow cytometry and histology.

### *Ex vivo* tumour flow cytometry and histology

For flow cytometry, 4T1 orthotopic breast tumours from two female BALB/c mice were stored in 10% DMSO in FBS and gradually cooled to −80 °C surrounded by RT isopropanol. Details of sample preparation and staining for pan macrophage marker F4/80, M1 marker CD80 and M2 marker CD206 can be found in Supplementary Methods. For immunofluorescence imaging, tumours were dissected, snap frozen, embedded and cut at 5 µm thickness before mounting on poly-lysine coated slides. Details surrounding further section preparation and staining for IBA-1 and F4/80 and analysis can be found in Supplementary Methods.

### PET/CT imaging and *ex vivo* biodistribution

Mice bearing 4T1 orthotopic breast tumours were anaesthetised as above before intravenous tail vein injection of 1) 10 µg DFO-IgG2b labelled with ~ 100 kBq [^89^Zr]Zr; 2) 10 µg DFO-anti-F4/80 labelled with ~ 200 kBq [^89^Zr]Zr; 3) 100 µg DFO-IgG2b labelled with ~ 800 kBq [^89^Zr]Zr; or 4) 100 µg DFO-anti-F4/80 labelled with ~ 1.4 MBq [^89^Zr]Zr in 130 µL 0.5% BSA/PBS (*n* = 5 per group). Static imaging, was conducted on a nanoPET/CT scanner (Mediso Medical Imaging Systems) 24 h after injection of the radiotracer (*n* = 3/group, see Supplementary Methods). Images were then reconstructed and analysed as per Supplementary Methods. Imaged animals were culled and dissected for *ex vivo *biodistribution after the scan, and data were combined with that of the two remaining non-imaged animals in each group. Dissected organs were weighed, and the radioactivity counted in a gamma counter (LKB Wallac, CompuGamma). The tissue uptake was calculated as the percentage of injected activity (IA) normalised to tissue weight (%IA/g).

For imaging with [^89^Zr]Zr-oxine-labelled cells, tumour-bearing mice were injected intravenously with 1 × 10^7^ [^89^Zr]Zr-oxine-labelled M0 macrophages (0.5–0.7 MBq). At 24-h post-injection, three mice were scanned as above.

### Statistical analysis

Data are presented as mean ± standard deviation. Statistical analysis was carried out using GraphPad Prism v9.1.0. Statistical significance was annotated as *, *p < *0.05; **, *p < *0.01; ***, *p < *0.001; and ****, *p < *0.0001. A one-way ANOVA was carried out for in vitro uptake studies. A two-way ANOVA with Sidak's multiple comparisons test was carried out for *ex vivo* biodistribution and PET image data sets, and a multiple unpaired *t-*test was carried out for *ex vivo *biodistribution studies involving 10 versus 100 μg datasets.

## Results

### Successful radiolabelling and specific receptor-mediated uptake of [^89^Zr]Zr-DFO-anti-F4/80

Anti-F4/80 mAb and its isotype control (IgG2b) were first conjugated to p-NCS-Bz-DFO and then radiolabelled with [^89^Zr]ZrCl_4_. [^89^Zr]Zr-DFO-anti-F4/80 and [^89^Zr]Zr-DFO-IgG2b were produced at specific activities of 9–11 and 16–18 MBq/mg, respectively (76–85% radiochemical yield), and successfully purified (> 95% radiochemical purity; Figure S3).

**Fig. 1:**
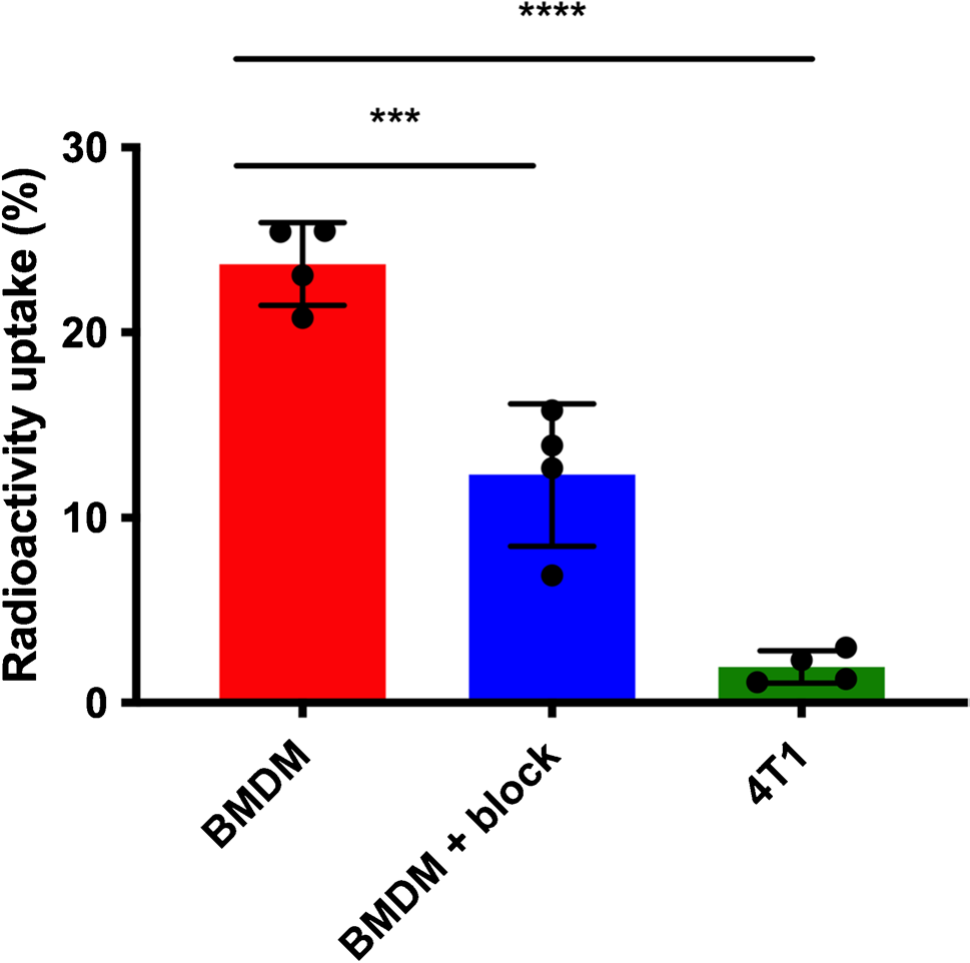
Receptor-specific uptake of [^89^Zr]Zr-DFO-F4/80 in cells. Uptake of [^89^Zr]Zr-DFO-anti-F4/80 in Bone Marrow-Derived Macrophages (BMDM) and 4T1 cells at 1 h post incubation. BMDMs were also co-incubated with [^89^Zr]Zr-DFO-anti-F4/80 and a blocking amount of unlabelled anti-F4/80 antibody (10 μg)

BMDMs, which express F4/80 (Figure S1), were used to determine binding and uptake of [^89^Zr]Zr-DFO-anti-F4/80. Purified [^89^Zr]Zr-DFO-anti-F4/80 was incubated with murine BMDMs; 23.1 ± 3.2% of the added [^89^Zr]Zr-DFO-anti-F4/80 bound to BMDMs. This amount decreased to 12.7 ± 1.7% (*p* = 0.0022) in the presence of excess DFO-anti-F4/80 (Fig. 1). Uptake of [^89^Zr]Zr-DFO-anti-F4/80 in 4T1 murine breast cancer cells, which do not express F4/80, was minimal, at 2.3 ± 1.2% (*p < *0.0001, Fig. 1).

### [^89^Zr]Zr-DFO-anti-F4/80 non-invasively images resident macrophages by PET/CT

**Fig. 2:**
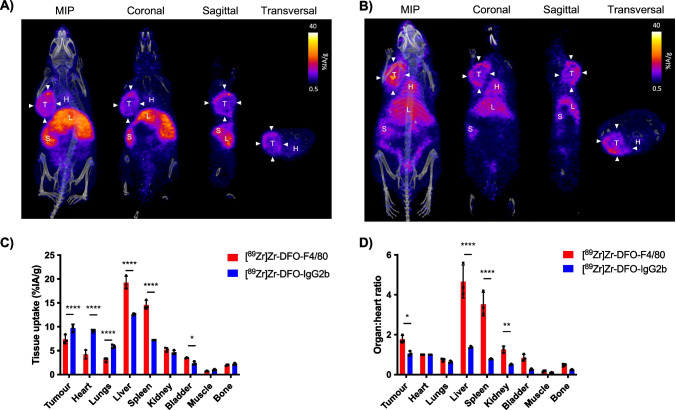
[^89^Zr]Zr-DFO-anti-F4/80 non-invasively images resident macrophages in the tumour, liver and spleen. PET/CT images of 4T1 tumour-bearing BALB/c mice (including Maximum Intensity Projections (MIPs)), 24 h post injection of 100 μg of (**A**) [^89^Zr]Zr-labelled DFO-anti-F4/80 and (**B**) [^89^Zr]Zr-labelled DFO-IgG2b. T: tumour (arrow heads), H: heart, L: liver, S: spleen. (**C**) PET image quantification of selected organs and tumours expressed as percentage injected activity per gram (%IA/g). (**D**) PET image quantification of selected organs and tumours with data presented as organ to heart ratios of radioactivity concentration

**Fig. 3:**
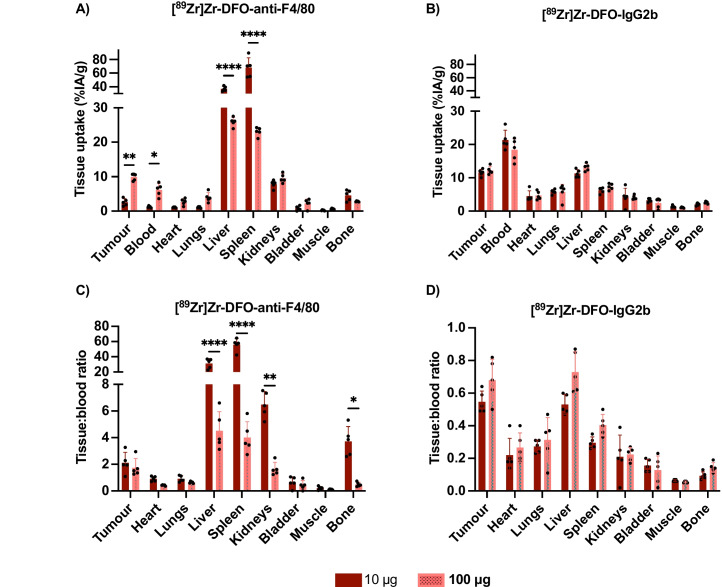
Injected antibody amount affects radiopharmaceutical biodistribution differently for [^89^Zr]Zr-DFO-anti-F4/80 and [^89^Zr]Zr-DFO-IgG2b. *Ex vivo* biodistribution data at 24 h post intravenous administration of [^89^Zr]Zr-DFO-anti-F4/80 and [^89^Zr]Zr-DFO-IgG2b at 10 μg (dark red) and 100 μg (dotted pink) immunoconjugate to female BALB/c mice bearing orthotopic 4T1 tumours. Data are presented as percentage injected activity per gram of tissue (%IA/g) (**A**, **B**) or as organ to blood ratios of radioactivity concentration (**C**, **D**)

[^89^Zr]Zr-DFO-anti-F4/80 and its isotype control, [^89^Zr]Zr-DFO-IgG2b, (0.8–1.4 MBq, 100 μg of immunoconjugate) were each administered to female BALB/c mice bearing orthotopic murine 4T1 breast tumours. At 24 h post-administration, PET/CT imaging and *ex vivo* biodistribution studies were undertaken (Figs. 2 and 3). Quantification of PET/CT images of mice injected with 100 μg of either [^89^Zr]Zr-labelled DFO-anti-F4/80 or DFO-IgG2b (Figs. 2A-B) indicated that, unexpectedly, there were lower concentrations of [^89^Zr]Zr-DFO-anti-F4/80 in the tumour than [^89^Zr]Zr-DFO-IgG2b (7.4 ± 0.9%IA/g versus 9.7 ± 0.8%IA/g respectively, *p < *0.0001, Fig. 2C). However, there was a significantly higher concentration of [^89^Zr]Zr-DFO-IgG2b in the heart – here used as a proxy for the blood pool – compared to [^89^Zr]Zr-DFO-anti-F4/80 (9.15 ± 0.3%IA/g versus 4.2 ± 0.9%IA/g respectively, *p < *0.0001, Fig. 2C).

Given the differential blood and tumour-associated radioactivity seen in both radioimmunoconjugates, we compared the *ratio* of ^89^Zr radioactivity in the tumour and the heart. The tumour-to-heart ratio measured 1.8 ± 0.2 for [^89^Zr]Zr-DFO-anti-F4/80 compared to 1.1 ± 0.1 for [^89^Zr]Zr-DFO-IgG2b (*p* = 0.0143, Fig. 2D). This led to a significantly greater tumour-to-background contrast in PET/CT images of mice administered with [^89^Zr]Zr-DFO-anti-F4/80 compared to those administered with [^89^Zr]Zr-DFO-IgG2b.

Significantly, for mice administered with [^89^Zr]Zr-DFO-anti-F4/80, higher ^89^Zr concentrations were observed in the liver (19.3 ± 1.3%IA/g), and spleen (14.6 ± 0.9%IA/g) than for [^89^Zr]Zr-DFO-IgG2b, which accumulated at 12.6 ± 0.2%IA/g in the liver (*p < *0.0001) and 7.2 ± 0.0%IA/g in the spleen (*p < *0.0001, Figs. 2A-C). Liver-to-heart and spleen-to-heart ratios were also higher for animals administered with [^89^Zr]Zr-DFO-anti-F4/80 compared to those administered with [^89^Zr]Zr-DFO-IgG2b (Fig. 2D). The liver and the spleen are known to contain reservoirs of macrophages [39].

*Ex vivo* quantification of the biodistribution of ^89^Zr radioactivity was consistent with PET/CT imaging quantification (Fig. 3).

### Antibody dose affects ^89^Zr-radiotracer biodistribution

Our prior findings showed that increasing the amount of receptor-specific [^111^In]In-anti-F4/80 antibody from 10 to 100 µg by effectively adding more non-radioactive anti-F4/80 decreased splenic and liver uptake, where macrophages are abundant, which in turn enhanced tumour uptake [37]. Here, we therefore also studied both 10 µg and 100 µg of either DFO-anti-F4/80 or DFO-IgG2b, each labelled with ^89^Zr. The *ex vivo *biodistribution was quantified at 1-day post-administration of radiotracer (Fig. 3). For mice administered with 10 μg of DFO-anti-F4/80 immunoconjugate, the tumour concentration of ^89^Zr measured 2.6 ± 1.0%IA/g at 1-day post-administration. This tumour concentration increased nearly five-fold to 9.9 ± 1.0%IA/g in mice administered with 100 μg of DFO-anti-F4/80 immunoconjugate (Fig. 3A). Conversely, animals administered with 10 μg of DFO-anti-F4/80 immunoconjugate had higher levels of ^89^Zr radioactivity in the liver and spleen. Additionally, the concentration of ^89^Zr in the blood pool measured 1.2 ± 0.2%IA/g with a 10 μg dose, compared to 6.2 ± 1.8%IA/g for a 100 μg dose (Fig. 3A). This biodistribution pattern is consistent with our hypothesis that DFO-anti-F4/80 and [^89^Zr]Zr-DFO-anti-F4/80 target endogenous, resident macrophages in well-known macrophage reservoirs, such as the spleen and liver, as well as TAMs.

In contrast, varying the amount of the control DFO-IgG2b from 10 µg to 100 μg had a minimal effect on tumour and normal tissue uptake. At an administered 10 μg radiolabelled immunoconjugate dose, 11.6 ± 1.0%IA/g was measured in tumours at 1-day post-administration; at 100 μg immunoconjugate dose, a similar ^89^Zr radioactivity concentration of 12.2 ± 1.2%IA/g was observed. Blood values for [^89^Zr]Zr-DFO-IgG2b at 10 µg was far higher than for [^89^Zr]Zr-DFO-anti-F4/80 and equalled 21.4 ± 2.9%IA/g. Furthermore, measured ^89^Zr concentrations in the blood pool, liver and spleen did not change significantly at these two different doses (*p* > 0.05, Fig. 3B). This suggests that the liver and spleen *do not* act as antigen sinks for [^89^Zr]Zr-DFO-IgG2b. Significantly, tissue-to-blood ratios changed for [^89^Zr]Zr-DFO-anti-F4/80 only (Figs. 3C-D).

### The 4T1 tumour model is abundant in macrophages

**Fig. 4:**
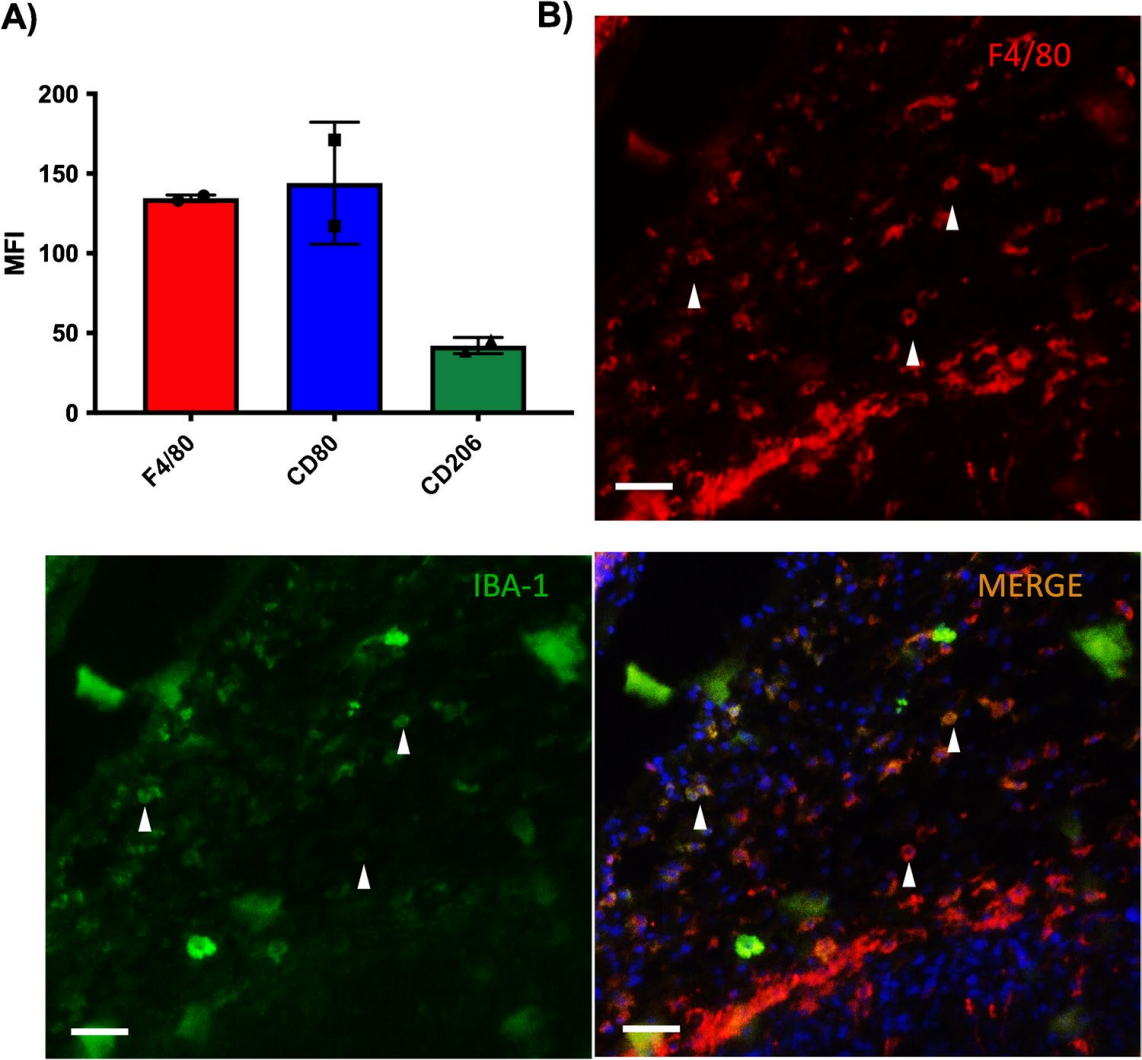
Presence of macrophages in the 4T1 tumour model. *Ex vivo* (**A**) flow cytometry (*n* = 2, 10,000 events/n) and (**B**) immunofluorescence for (A) F4/80, CD80 and CD206, or (B) DAPI (blue), F4/80 (red) and IBA-1 (green) in 4T1 tumour samples. MFI: mean fluorescence intensity (A). Arrow heads showcase examples of macrophages that are co-stained for IBA-1 and F4/80. Scale bar is 50 μm

*Ex vivo* flow cytometry and immunofluorescence staining of tumour tissue confirmed the presence of F4/80-positive macrophages in the tumour microenvironment (Fig. 4). These macrophages were also positive for CD80 and CD206 (Fig. 4A), which are markers for M1 and M2 macrophages, respectively, as well as IBA-1, a pan-macrophage marker (Fig. 4B).

### Characterisation and biodistribution of [^89^Zr]Zr-labelled macrophages

**Fig. 5:**
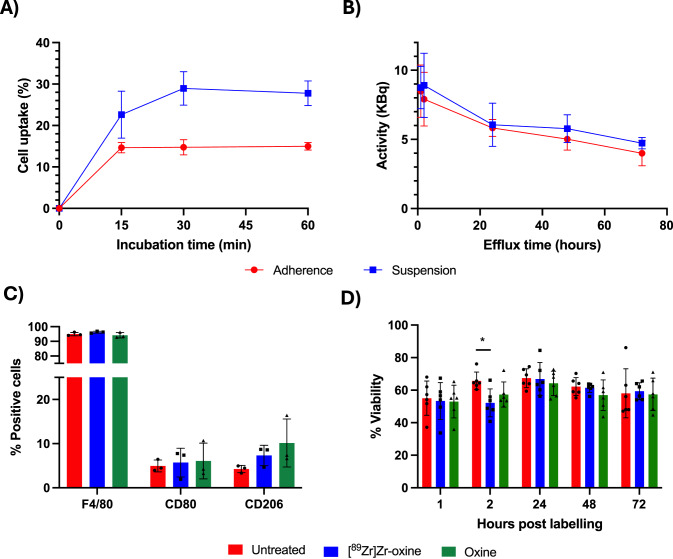
Radiolabelled M0 macrophages retain significant [^89^Zr]Zr radioactivity over time and [^89^Zr]Zr-radiolabelling does not affect key receptor expression or viability. (**A**) *In vitro *uptake of [^89^Zr]Zr-oxine in macrophages when adhered or suspended, over time. (**B**) [^89^Zr]Zr retention from macrophages labelled in suspension versus adherent. *N* = 3–5/group. (**C**) Number of cells positive for F4/80, CD80, and CD206 in M0 macrophages incubated with and without [^89^Zr]Zr-oxine or oxine alone, as assessed by flow cytometry. (**D**) Macrophage viability between untreated, [^89^Zr]Zr-oxine- and oxine alone-labelled macrophages that were scraped in suspension when radiolabelled. *n* = 3–6 per group

Murine M0 BMDMs were radiolabelled with [^89^Zr]Zr-oxine at 300 kBq per 10^6^ BMDMs (Figure S4, [21]). When adherent, macrophage labelling efficiency was 15.0 ± 0.9% of added [^89^Zr]Zr-oxine after 60 min incubation in serum-free RPMI-1640 (Fig. 5A), reaching specific activities of 45.0 ± 2.7 kBq per 10^6^ BMDMs. When macrophages were in suspension, cell radiolabelling yields increased to 27.8 ± 3.0% after 15 min incubation, achieving 83.4 ± 9 kBq per 10^6^ BMDMs. Once radiolabelled for one hour, efflux was unaltered between macrophages that had been radiolabelled when adherent or in suspension (*p* = 0.1407, Fig. 5B), retaining 36.7 ± 0.3% and 39.5 ± 1.2% in cells, respectively, over 72 h.

Cells labelled in suspension received a higher absorbed radiation dose during incubation, with a maximum absorbed dose of 3.31 Gy over 72 h, compared with 2.95 Gy for cells labelled whilst adherent (Table S1, Figure S5). Radiolabelling of M0 macrophages with [^89^Zr]Zr-oxine for 15 min did not affect macrophage phenotype as F4/80, CD80 or CD206 remained unaltered (Fig. 5C). Macrophage viability when radiolabelled remained largely unaffected (Figs. 5D, S6). Only at the 2-h timepoint was a significant decrease in viability observed; the viability of macrophages radiolabelled in suspension was 52.2% ± 8.6% versus 65.7% ± 5.4% for unlabelled macrophages at the same timepoint (Fig. 5D).

**Fig. 6:**
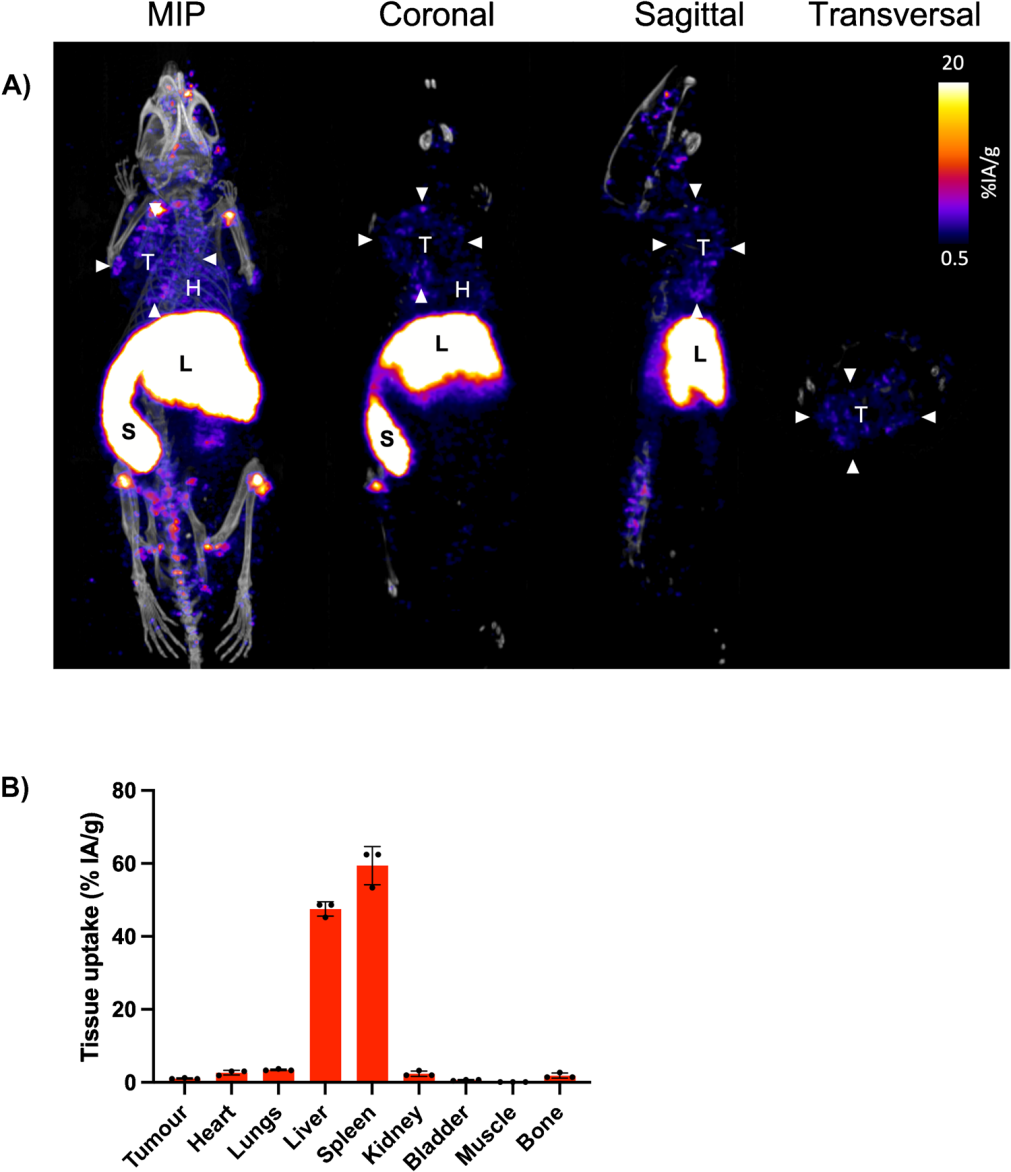
[^89^Zr]Zr-labelled macrophages localise to the liver and spleen with some uptake in tumour. (**A**) Maximum Intensity Projection (MIP) and PET/CT images of 4T1 tumour-bearing BALB/c mice, 24 h post intravenous injection of 1 × 10^7^ [^89^Zr]Zr-labelled macrophages. T: tumour (arrow heads), H: heart, L: liver, S: spleen. Image intensity was set to enable the tumours to be visualised. (**B**) PET image quantification of selected organs and tumours. Knee values were used to represent bone/bone marrow

Next, mice bearing 4T1 tumours were intravenously administered 1 × 10^7^ [^89^Zr]Zr-labelled M0 macrophages. PET/CT imaging was then conducted to determine whether macrophage biodistribution could be tracked. At 24 h post-injection, [^89^Zr]Zr-labelled macrophages were primarily localised in the liver (47.5 ± 2.0%IA/g) and spleen (59.4 ± 5.2%IA/g), with some accumulation in the tumour (1.1 ± 0.2%IA/g). [^89^Zr]Zr was also seen in the bone/bone marrow (1.9 ± 0.7%IA/g, Fig. 6). *Ex vivo *biodistributions confirmed similar uptake trends (Figure S7).

## Discussion

Traditionally, autologous leukocytes radiolabelled with [^111^In]In(oxine) have been clinically used to determine sites of inflammation or infection using γ-scintigraphy [40]. Macrophages have previously also been tracked preclinically using ^18^F-labelled superparamagnetic iron oxine nanoparticles for PET/MRI or [^125^I]I-PKH-95 for SPECT imaging [41, 42], and are also being investigated clinically using [^68^ Ga]Ga-NOTA-anti-MMR-VHH2 (NCT04168528). Here, we have developed two complementary methods for tracking macrophages using ^89^Zr PET imaging, which could enable highly sensitive and longitudinal quantification of macrophage migration and tumour accumulation in clinical disease settings. We postulate that long-term tracking of macrophages using [^89^Zr]-PET would reveal useful information about changes in macrophage distribution during the progression of many diseases and in response to therapies. Indeed, the short-lived PET tracer, *(R)-*[^11^C]PK11195, which targets macrophages, has shown such utility in rheumatoid arthritis, in the context of both disease progression and response to therapy [43, 44]. To highlight the potential of these methods, we applied both direct and indirect methods of ^89^Zr macrophage cell imaging in a murine syngeneic, orthotopic breast cancer model. PET imaging with [^89^Zr]Zr-DFO-anti-F4/80 enabled identification of resident macrophages in the tumour, as well as the liver and spleen. PET imaging with [^89^Zr]Zr-labelled macrophages enabled quantification of ^89^Zr macrophage migration from the blood pool to tumour, liver and spleen over the 24-h imaging protocol.

We have previously observed that administration of low amounts of an ^111^In-radiolabelled anti-F4/80 antibody radiotracer (10 µg) resulted in high spleen and liver uptake and low tumour accumulation [37]. However, at higher amounts of anti-F4/80 antibody (100 μg), the relative spleen and liver uptake decreased, whilst the relative tumour uptake increased. High concentrations of macrophages reside in the spleen and liver, and it is likely that at low levels of administered anti-F4/80 antibody, the majority of (labelled and unlabelled) immunoconjugate is specifically taken up by splenic macrophages. However, at high levels of administered anti-F4/80 antibody, receptor-specific binding to splenic macrophages nears saturation, making more radiolabelled anti-F4/80 immunoconjugate available to target other, more diffuse populations of macrophages, including TAMs [37]. Here, the *ex vivo *biodistribution studies comparing different amounts (10 and 100 µg) of each of the immunoconjugates, DFO-IgG2b and DFO-anti-F4/80, further highlighted the influence of liver- and spleen-resident macrophages as antigen sinks [37, 45], consistent with these and other prior studies that have developed alternative macrophage-specific tracers [3, 15, 24, 37, 46]. Furthermore, by increasing the immunoconjugate regime from 10 to 100 µg [^89^Zr]Zr-labelled DFO-anti-F4/80, we demonstrated that saturation of these sinks increased blood residency time of [^89^Zr]Zr-DFO-anti-F4/80, thereby increasing bioavailability and likely contributing to the observed increased uptake in TAMs of tumour tissue. These comparisons also confirmed that [^89^Zr]Zr-DFO-anti-F4/80 retained specificity for the F4/80 receptor and therefore macrophages. At 24 h post injection, the tumour-to-blood ^89^Zr radioactivity concentration ratio was significantly higher for [^89^Zr]Zr-DFO-anti-F4/80 at 100 µg than for [^89^Zr]Zr-DFO-IgG2b, with high tumour contrast also observed in PET images at the 100 µg amount. Localisation of [^89^Zr]Zr-DFO-IgG2b in tumour tissue was likely primarily perfusion-mediated.

As well as pursuing an approach to visualise TAMs already *in situ*, we also utilised the one-step, kit-based [^89^Zr]Zr-oxine method [21] to radiolabel BMDMs and quantify their biodistribution 24 h after intravenous administration. This kit-based [^89^Zr]Zr-oxine method is suitable for labelling BMDMs, with significant radiochemical yields achieved, alongside BMDM retention of ^89^Zr radioactivity. ^89^Zr-labelled BMDMs showed resistance to radiation absorbed doses up to 3.31 Gy, with no *in vitro* changes in cell surface receptor expression or viability observed in *in vitro *studies. Here, the accumulation of [^89^Zr]Zr-labelled macrophages in 4T1 murine breast cancer tumours was 1.1 ± 0.2%IA/g at 24 h post-injection. In previous studies, we demonstrated the co-localisation of radioactive signals with cells in tissues such as the spleen, liver, and tumour using [^89^Zr]Zr-oxine-labelled T-cells [22]. Here, since the biodistribution of free [^89^Zr]Zr-oxine differs from that of intact cells labelled with [^89^Zr]Zr-oxine, and because free [^89^Zr]Zr-oxine typically exhibits higher uptake levels in the heart, bone, and lungs at 24 h post-intravenous injection, it can be concluded that the ^89^Zr signals visualised and measured here originate from radiolabelled macrophages rather than free [^89^Zr]Zr-oxine or ^89^Zr [20, 47–49].

Advantages and disadvantages exist for both “indirect” receptor-targeted imaging approaches that visualise immune cells and “direct” imaging approaches utilising radiolabelled immune cells. For example, receptor-targeted imaging, through repeated radiotracer administration and imaging procedures can image resident cells over longer periods of time. However, this method tracks the biodistribution of the radiotracer rather than the cells themselves, with signal resulting from tracer accumulation in non-target tissues and incorporates radiotracer excretion, as also seen for other radiotracers. Here, [^89^Zr]Zr-DFO-anti-F4/80 enabled imaging of tumour-resident macrophages. As F4/80 is a marker of murine and not human macrophages, however, its applicability as a research tool will remain limited to the preclinical setting. Nonetheless, the visualisation of murine macrophages could provide a useful tool to test the efficacy of novel therapeutics targeting TAM populations. Equally, the approach used here for [^89^Zr]Zr-DFO-anti-F4/80 could be applied to human pan macrophage markers, such as CD68, or TAM-specific markers such as CD206.

In contrast to receptor-targeted imaging, “direct” cell imaging with [^89^Zr]Zr-labelled macrophages could facilitate studies that explore the migration of macrophages to tumours at different stages of development. It does only enable macrophages to be tracked however for as long as the decay properties of the ^89^Zr radionuclide will allow. For ^111^In and ^89^Zr, this is between 1–2 weeks, although the advent of total body PET is likely to elongate the time frame for ^89^Zr [50]. Additionally, this method only allows for the administered radiolabelled cells to be imaged – resident and endogenous immune cells remain “silent” during imaging procedures. Lastly, dissociation or leaching of the radionuclide from the cells dilutes the signal and leads to off-target accumulation.

By combining the techniques as we have here, complementary insights were obtained, ameliorating limitations of employing only a single imaging method. For example, receptor-targeted imaging indicates there are likely significant populations of macrophages already resident at the tumour site. Equally, direct PET tracking of macrophages evidenced the migration of macrophages from the blood pool to the liver and spleen, and significantly, to the tumour. The 24 h timeframe employed here is likely insufficient to track the migration of macrophages from tissue ‘reservoirs’ to the tumour.

## Conclusion

For the first time, both direct and indirect PET/CT approaches have been applied and compared for imaging F4/80-positive BMDMs in a syngeneic, orthotopic breast cancer mouse model. This has included the development of two novel radiotracers, namely [^89^Zr]Zr-DFO-anti-F4/80 targeted to the F4/80 receptor and *ex vivo-*labelled [^89^Zr]Zr-labelled macrophages. Both approaches evidence significant infiltration of macrophages in breast cancer, a disease in which tumour-associated macrophages are critical in determining disease progression within the tumour microenvironment.

A high level of resident TAMs was visualised in tumours by [^89^Zr]Zr-DFO-anti-F4/80 PET/CT imaging whilst [^89^Zr]Zr-labelled M0 macrophages enabled quantification of macrophage migration, from the blood pool to organs and tissues including liver, spleen and tumour. Macrophage phenotype proved mostly unaffected by ^89^Zr-labelling in our experimental timeline. Ultimately, both direct and indirect PET imaging of macrophages could prove a useful in vivo tool to understand tumour evolution and interrogate cancer therapies in the context of macrophages in the tumour microenvironment.

## Supplementary Information

Below is the link to the electronic supplementary material.

Supplementary file1 (DOCX 1070 KB)

## Data Availability

Data is available upon reasonable request to the corresponding author.
