# Supplementary material for: Tracking macrophages by direct and indirect 89Zr PET imaging
Source: Eur J Nucl Med Mol Imaging. 2026 Feb 13;53(6):4130–42. doi: 10.1007/s00259-025-07752-8 (PMC13121358; doi:10.1007/s00259-025-07752-8)
Supplement: Supplementary file 1 — Supplementary file1 (DOCX 1070 KB) [file 259_2025_7752_MOESM1_ESM.docx]

**SUPPLEMENTAL DATA**

**Supplemental methods**

**Antibody conjugation with DFO**

Conjugation procedures were carried out in metal-free conditions. First, Vivaspin™ ultrafiltration spin columns (2.5 mL 30 kDa molecular weight cut-off polyethersulfone (PES) membranes, Sartorius, UK) were equilibrated with 0.1 M N-2-hydroxyethylpiperazine-N'-2-ethanesulfonic acid (HEPES) buffer (pH 8.9, Merk/Sigma Aldrich). Rat anti-F4/80 or IgG2b (Abcam) were pooled into separate Vivaspin™ columns and centrifuged at 1,780 *g* and washed with 0.1 M HEPES (pH 8.9). The Vivaspin™ column was then inverted and centrifuged at 500 *g* for 5 minutes, the antibodies retrieved, and antibody concentrations measured using the Nanodrop 2000 (ThermoFisher Scientific).

Antibodies (6-7 mg) were incubated with p-isothiocyanatobenzyl-desferrioxamine (p-NCS-Bz-DFO in DMSO) at a 10:1 molar equivalent ratio of p-NCS-Bz-DFO:antibody at 37°C for 30 minutes followed by 16-hour incubation at room temperature (RT). Unconjugated p-NCS-DFO was separated from DFO-anti-F4/80 or DFO-IgG2b using pre-equilibrated PD10 columns (Cytiva) with 0.1 M ammonium acetate (pH 5.5), then 1% bovine serum albumin, and finally 0.1 M ammonium acetate (pH 5.5) again). Eluate was collected in 20 fractions (250 – 500 µL) with 0.1 M ammonium acetate (pH 5.5). The four fractions with the highest antibody concentrations were pooled and reconcentrated by Vivaspin™ columns. Antibody concentration protocol was as above.

**Isolation of bone marrow-derived monocytes and differentiation to M0 macrophages**

Femurs and tibias from female BALB/c mice were kept into RPMI-1640 (Invitrogen/Thermo) augmented with 10% foetal bovine serum, 1% penicillin/streptomycin (Invitrogen) and 1% L-glutamine (Invitrogen) (full growth medium). The bone marrow was flushed with full growth medium in class II tissue culture hood and isolated by centrifugation at 500 *g* for 5 minutes. The bone marrow pellet was resuspended in red blood cell lysis buffer for 5 minutes at RT before being pelleted again, resuspended in full growth medium and filtered through a 70 µm cell strainer. Cells were plated at 1x10^6^ cells/mL in full growth medium augmented with 10 ng/mL murine macrophages-colony stimulating factor (mM-CSF) for 72 hours at 37 °C in a humidified 5% CO_2_ atmosphere to form M0 macrophages (bone marrow-derived macrophages, BMDM). Each mouse yielded on average 2x10^7^ cells after isolation and differentiation to M0 macrophages.

**Immunofluorescence staining and imaging**

BMDMs were grown on coverslips at 5x10^4^ cells per well in 24-well plates in 1 mL medium and cultured for 72 hours. Medium was then removed, and cells were fixed with 500 µL 4% paraformaldehyde, 2% sucrose solution (pH 7.2) at RT for 10 minutes. Cells were then washed with PBS and permeabilised with 100 µL 0.2% Triton X-100 in PBS for 2.5 minutes at RT. Cells were then washed as before and blocked with 2 % BSA in PBS for 30 minutes at RT. After this time, cells were incubated using rabbit-anti-IBA-1 primary antibody (Abcam, 1:100) overnight at 4°C. Cells were then washed before being incubated with 1:200 FITC anti-rabbit secondary antibody (Abcam) and 1:200 PE anti-F4/80 antibody (BioLegend) for 1 hour at RT. Cells were then washed and mounted on glass slides using VectaShield with nuclear stain, DAPI, and imaged (EVOS M5000 Imaging System; Invitrogen). Negative controls consisted of unstained slides and slides stained with secondary antibody only, which showed no staining.

***In vitro* flow cytometry**

Cells were harvested and pelleted by centrifugation at 500 *g* for 3 minutes, washed in PBS and fixed in 4% paraformaldehyde for 10 minutes at RT. After two further PBS washes, cells were resuspended to 1 x 10^6^ cells/mL in magnetic-activated cell sorting MACS buffer. Cells were then stained with anti-F4/80-PE in 0.5% BSA/PBS (1:200, Biolegend) at 4°C for 30 min and acquired with a FACS Melody (BD Biosciences). Flow analysis was carried out using FlowJo v10.7.

***Ex vivo* tumour flow cytometry and microscopy**

For flow cytometry, 4T1 orthotopic breast tumours from female BALB/c mice were stored in 10% DMSO in FBS and gradually cooled to -80°C surrounded by isopropanol. Each tumour was then digested using 50 mL RPMI-1640, 50 μg Collagenase 1 (1 mg/mL stock) and 5 μg DNAase I (0.1 mg/mL stock) for 1 hour at 37°C shaking at 500 rpm. The tumour digest was then filtered through a 70 µm filter, washed with RPMI-1640, and centrifuged at 500 *g* for 5 minutes at 4°C. The pellet was resuspended, washed in RPMI-1640 and fixed in 4% paraformaldehyde for 10 minutes at RT. After two further PBS washes, cells were resuspended to 60 x 10^6^ cells/mL in sterile PBS containing 2 mM EDTA and 1% FBS. Cells were then stained and analysed for F4/80, CD80 and CD206 as above. Antibody dilutions were 1:200, 1:100 and 1:100 for anti-F4/80-PE, anti-CD80-APC, and anti-CD206-AlexaFluor-488 in 0.5%BSA/PBS (all Biolegend), respectively.

For immunofluorescence imaging, tumours were dissected, snap frozen in isopentane on dry ice, embedded in OCT (VWR) and cut at 5 µm thickness using a Cryostat MNT (SLEE) before mounting on poly-lysine coated slides. After sectioning, slides were airdried for 30 minutes and fixed using 4% paraformaldehyde, 2% sucrose solution (pH 7.2) at RT for 10 minutes. Sections were then washed using PBS and blocked using 1% horse serum for 30 minutes at RT after which rabbit-anti-IBA1 (1:200) was incubated at 4°C overnight. Sections were then washed before incubating with FITC anti-rabbit (1:200) and anti-murine F4/80-PE for 1 hour at RT. Sections were then washed again and mounted using VectaShield with DAPI. Images were taken using the EVOS M5000 Imaging System (Invitrogen).

**Cellular dosimetry**

Radiation absorbed dose to macrophages, labelled in suspension or in adherence, was calculated for each time point. The distribution of activity was approximated by a compartmental model (Figure S2).

The differential equations representing the activity time-course in the medium and in the cells during the labelling phase and incubation phases are given in Equations S1 and S2 (labelling), S3 and S4 (incubation).

$$\begin{aligned} \frac{dA_{M}\left( t \right)}{dt}=-K_{1}A_{M}\left( t \right)+k_{2}A_{C}\left( t \right) \#S1 \end{aligned}$$

$$\begin{aligned} \frac{dA_{C}\left( t \right)}{dt}=K_{1}A_{M}\left( t \right)+k_{2}A_{C}\left( t \right) \#S2 \end{aligned}$$

$$\begin{aligned} \frac{dA_{M}\left( t \right)}{dt}=k_{2}A_{C}\left( t \right) \#S3 \end{aligned}$$

$$\begin{aligned} \frac{dA_{C}\left( t \right)}{dt}=-k_{2}A_{C}\left( t \right) \#S4 \end{aligned}$$

where *A* represents the activity in a compartment at time *t*, *M* and *C* refer to medium and cells, *K1* represents the rate of uptake from medium to cells and *k2* represents the rate of efflux from cells to medium.

These equations were solved analytically to calculate the time-integrated activity in cells was calculated over the labelling and incubation periods and included the calculated rate of efflux. The activity in the cells at the end of the labelling period was used to determine the internalisation rate. The following assumptions were used: (i) during labelling, [^89^Zr]Zr-oxine added to the medium at t = 0 is internalised into the cells with a rate constant, *K1*; (2) within the cell, the labelled complex is broken down and free ^89^Zr undergoes efflux with rate constant k2; (iii) at the end of the labelling period, the labelling reaction was ended by removing the radioactive medium; (iv) for the incubation period, cells are incubated in fresh, non-radioactive medium. Activity in the cells undergoes efflux with rate constant *k2*.

The time-integrated activity was multiplied by S-values for self-dose and cross-dose. The S-values were determined using the MIRDcell software, assuming a 10 µm macrophage radius and 100% internalisation of the [^89^Zr]Zr-oxine. Cross-dose S-values were determined by assuming that cells were uniformly distributed in a 2D monolayer throughout the course of incubation. Only self-dose was considered during the labelling process.

**PET/CT imaging, reconstruction and analysis**

Static imaging was conducted on a nano/CT scanner (Mediso Medical Imaging Systems, Budapest, Hungary) 24 hours after tail vein intravenous injection of the radiotracer (n=3/group). Animals were anaesthetised as above and transferred to an air-heated single-mouse bed; respiration was monitored throughout. A scout CT scan was conducted to ensure correct position of the animal in the field of view followed by a 45-minute PET acquisition scan with a 400-600 keV energy window, 4 ns coincidence window and 1-5 coincidence mode. Finally, a full helical CT scan was acquired with 55 kVp X-ray, 600 ms exposure time and 180 projections.

Image reconstruction of PET/CT was carried out on Nucline version 2.01.020 (Mediso Medical Imaging System). Monte Carlo-based Tera-Tomo 3D PET reconstruction was performed with 1-3 coincidence mode, 4 iterations and 6 subsets. Isotropic voxel size was set at 0.4 mm^3^, images were corrected for scatter, attenuation, and decay corrected to the time of injection 24 hours before the scan.

Region of interest quantification analysis of reconstructed images was carried out on VivoQuant (Invicro Inc). Post processing was carried out to convert MBq values to injected activity per gram (%IA/g), after which regions of interest were drawn for all target and reference organs and the amount of radioactivity present in the regions of interest extracted and plotted.

**Supplemental figures**

**Figure S1. A)** Gating of live cells separating using side scatter area (SSC-A) and forward scatter area (FCS-A). **B)** Gating of single cells using forward scatter area (FCS-H, Y-axis) and forward scatter area (FCS-A, X-axis) to exclude doublets and aggregates. This gated population was used to derive further flow cytometry analysis graphs. Histograms of cell count for unstained (**C)** and PE-anti-F4/80-stained **(D)** M0 macrophages. **(E)** Immunofluorescence imaging of IBA-1 and F4/80 expressions in bone marrow-derived macrophages (BMDM). Scale bar is 50 μm.


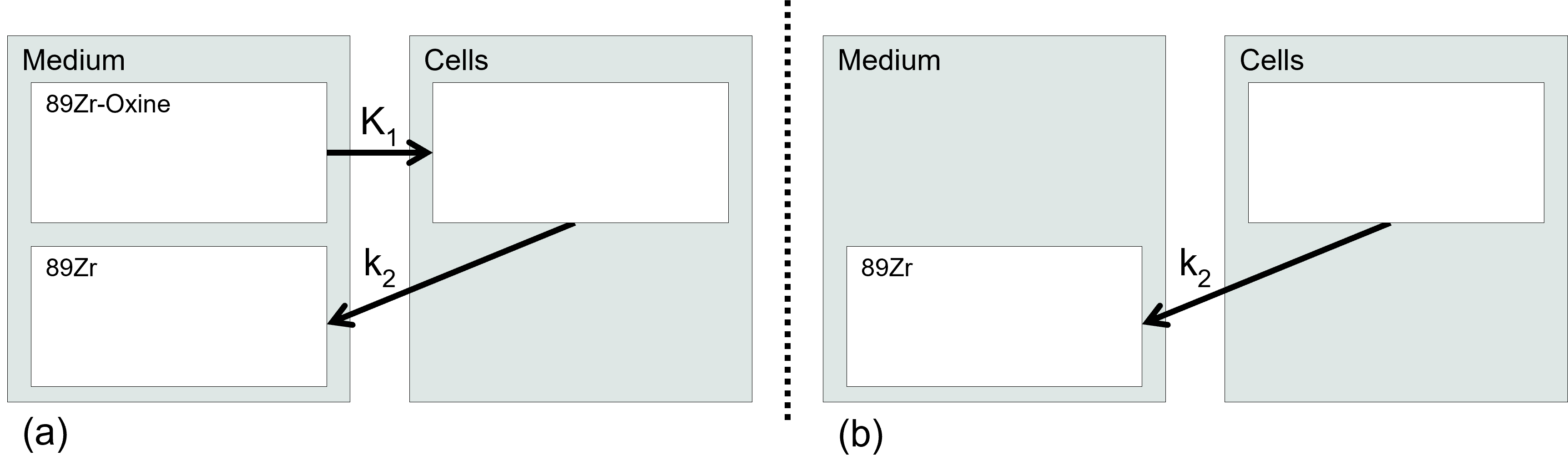


**Figure S2:** Schematic representations of compartmental models used to represent distribution of activity in (a) labelling and (b) incubation phases.

**
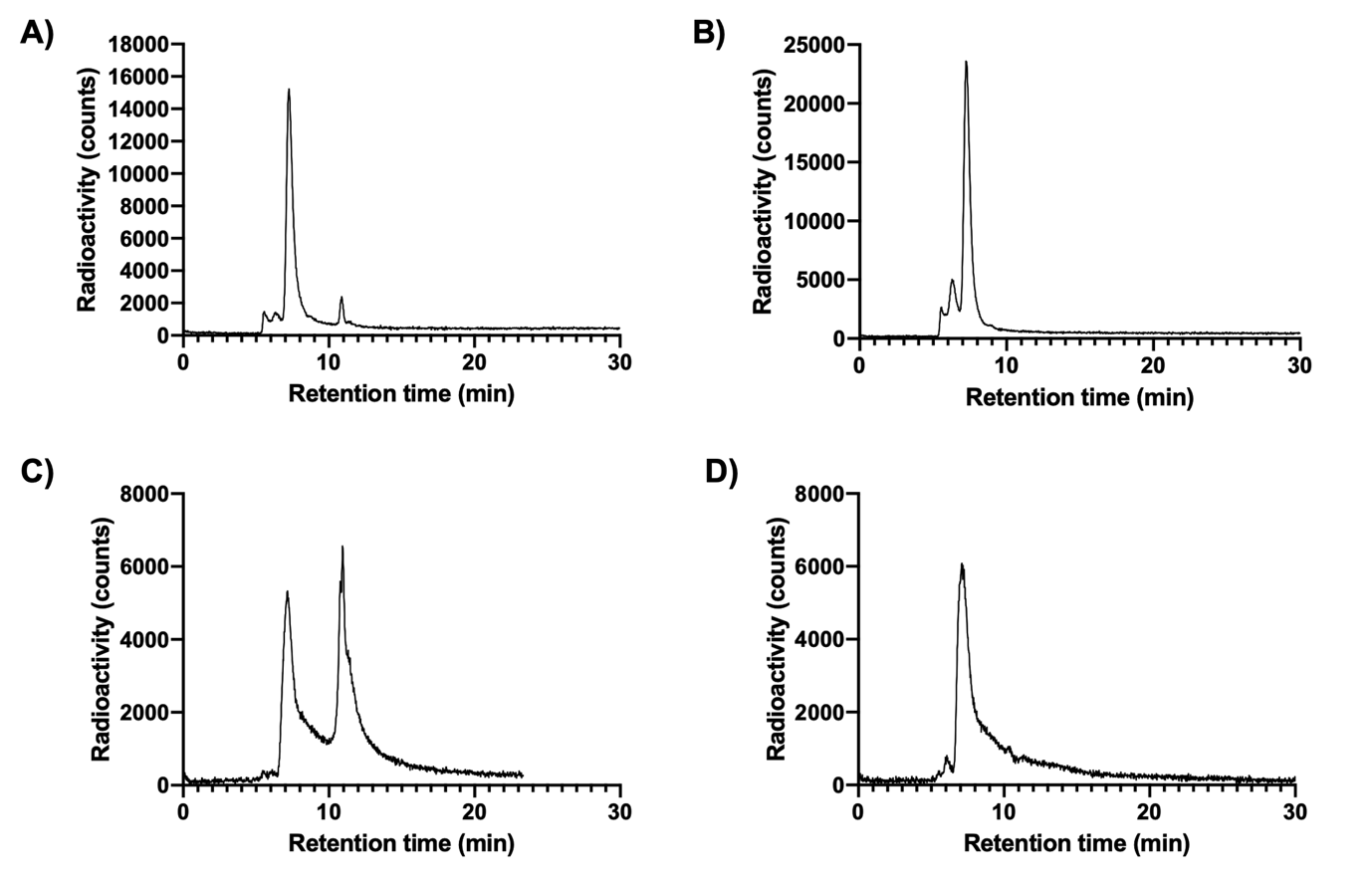
**

**Figure S3.** Size-exclusion HPLC radiochromatograms of (A, B) [^89^Zr]Zr-DFO-F4/80 and (C, D) [^89^Zr]Zr-DFO-IgG2b before (A, C) and after (B, D) PD-10 purification. Retention times of radiolabelled antibodies and free ^89^Zr were 7.5 and 12.5 minutes, respectively.

**Figure S4.** iTLC chromatogram showing a typical quality control run after [^89^Zr]Zr-oxine synthesis using the kit described herein. Bkg1 refers to the background radiation level to obtain a baseline for the readout. Region 1 refers to the location where the sample was spotted and contains unreacted/unconjugated ^89^Zr-oxalate. Region 2 shows the [^89^Zr]Zr-oxine peak. The associated areas under the curve of regions 1 and 2 were used to calculate radiochemical yield.


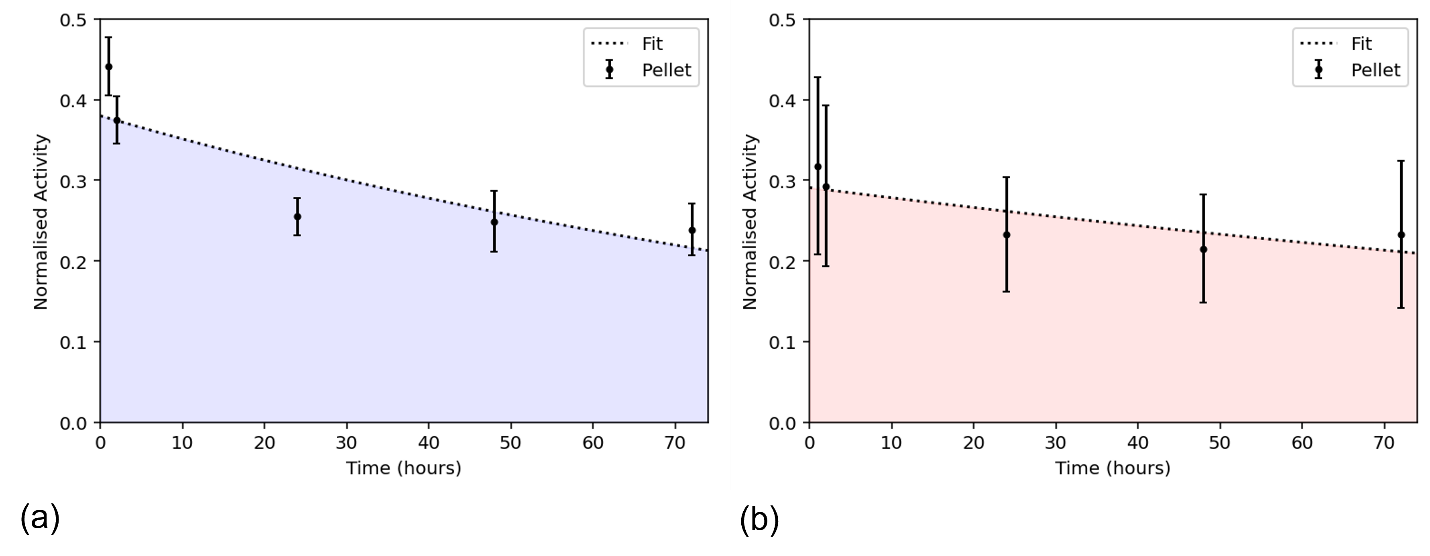
 **Figure S5.** Activity retained in macrophages over varying incubation times in cells labelled in suspension (a) and in adherence (b), decay corrected to end of labelling period.

**
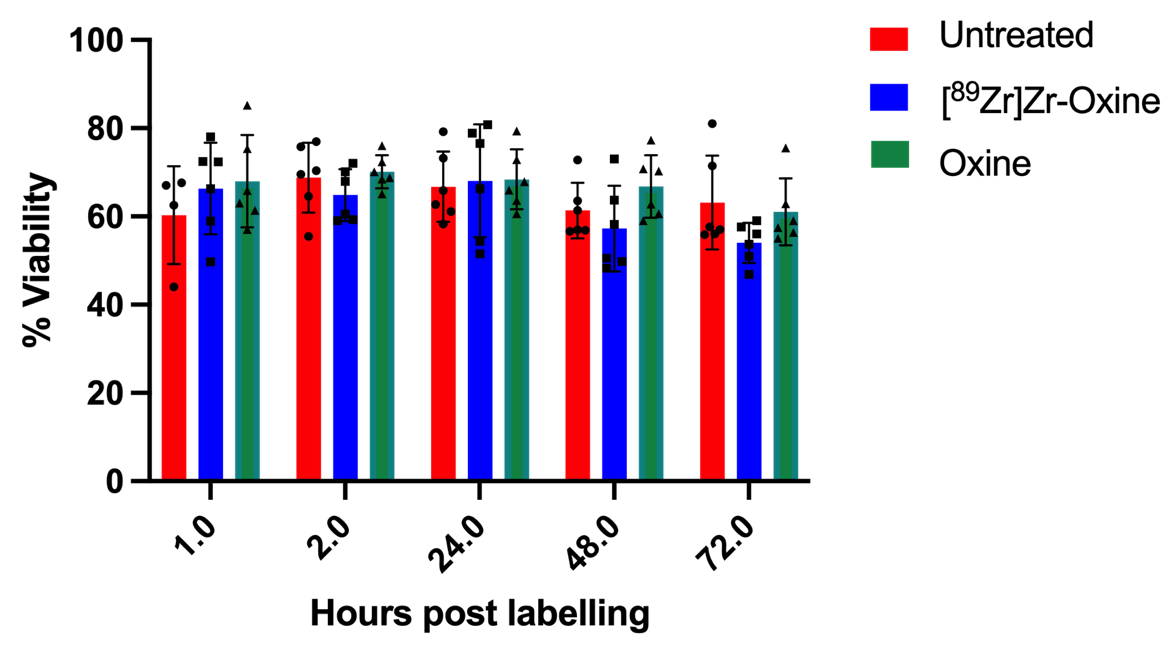
**

**Figure S6.** Macrophage viability as measured by trypan blue exclusion between untreated, [^89^Zr]Zr-oxine- and oxine alone-labelled macrophages that were adherent when labelled. N=6 per group.


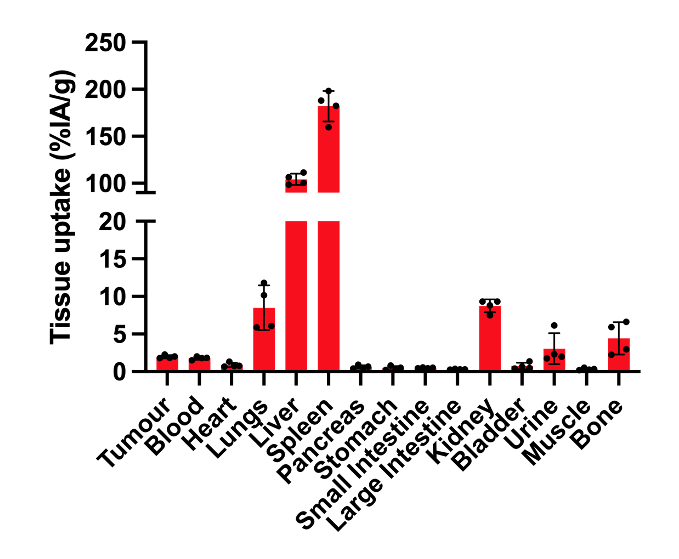


**Figure S7.** *Ex vivo* biodistribution data of [^89^Zr]Zr-labelled macrophages at 24 hours post injection into 4T1 tumour-bearing BALB/c mice. Data is expressed as percentage injected activity per gram (%IA/g).

**Supplemental table**

***Table S1.*** *Dosimetry of macrophage labelling and incubation when adherent or in suspension.*

|  | Suspension |  |  |  | Adherent |  |  |  |
| --- | --- | --- | --- | --- | --- | --- | --- | --- |
| Time (h) | Labelling Self-Dose (Gy) | Incubation Self-Dose (Gy) | Incubation Cross-Dose (Gy) | Total Dose (Gy) | Labelling Self-Dose (Gy) | Incubation Self-Dose (Gy) | Incubation Cross-Dose (Gy) | Total Dose (Gy) |
| 1 | 0.02 | 0.08 | 0.00 | 0.10- | 0.02 | 0.06 | 0.00 | 0.08 |
| 2 | 0.02 | 0.15 | 0.00 | 0.17 | 0.02 | 0.12 | 0.00 | 0.14 |
| 24 | 0.02 | 1.53 | 0.02 | 1.57 | 0.02 | 1.29 | 0.01 | 1.31 |
| 48 | 0.02 | 2.55 | 0.04 | 2.61 | 0.02 | 2.22 | 0.02 | 2.26 |
| 72 | 0.02 | 3.25 | 0.05 | 3.31 | 0.02 | 2.91 | 0.03 | 2.95 |
